# An improved sparse representation model with structural information for Multicolour Fluorescence *In-Situ *Hybridization (M-FISH) image classification

**DOI:** 10.1186/1752-0509-7-S4-S5

**Published:** 2013-10-23

**Authors:** Jingyao Li, Dongdong Lin, Hongbao Cao, Yu-Ping Wang

**Affiliations:** 1 Department of Biomedical Engineering, Tulane University, New Orleans, LA, 70118 USA; 2 Department of Biostatistics & Bioinformatics, Tulane University, New Orleans, LA, 70112 USA; 3 Shanghai University for Science and Technology, Shanghai, 200090, China

## Abstract

**Background:**

Multicolour Fluorescence *In-Situ *Hybridization (M-FISH) images are employed for detecting chromosomal abnormalities such as chromosomal translocations, deletions, duplication and inversions. This technique uses mixed colours of fluorochromes to paint the whole chromosomes for rapid detection of chromosome rearrangements. The M-FISH data sets used in our research are obtained from microscopic scanning of a metaphase cell labelled with five different fluorochromes and a DAPI staining. The reliability of the technique lies in accurate classification of chromosomes (24 classes for male and 23 classes for female) from M-FISH images. However, due to imaging noise, mis-alignment between multiple channels and many other imaging problems, there is always a classification error, leading to wrong detection of chromosomal abnormalities. Therefore, how to accurately classify different types of chromosomes from M-FISH images becomes a challenging problem.

**Methods:**

This paper presents a novel sparse representation model considering structural information for the classification of M-FISH images. In our previous work a sparse representation based classification model was proposed. This model employed only individual pixel information for the classification. With the structural information of neighbouring pixels as well as the information of themselves simultaneously, the novel approach extended the previous one to the regional case. Based on Orthogonal Matching Pursuit (OMP), we developed simultaneous OMP algorithm (SOMP) to derive an efficient solution of the improved sparse representation model by incorporating the structural information.

**Results:**

The p-value of two models shows that the newly proposed model incorporating the structural information is significantly superior to our previous one. In addition, we evaluated the effect of several parameters, such as sparsity level, neighbourhood size, and training sample size, on the of the classification accuracy.

**Conclusions:**

The comparison with our previously used sparse model demonstrates that the improved sparse representation model is more effective than the previous one on the classification of the chromosome abnormalities.

## Background

**Figure 1:**
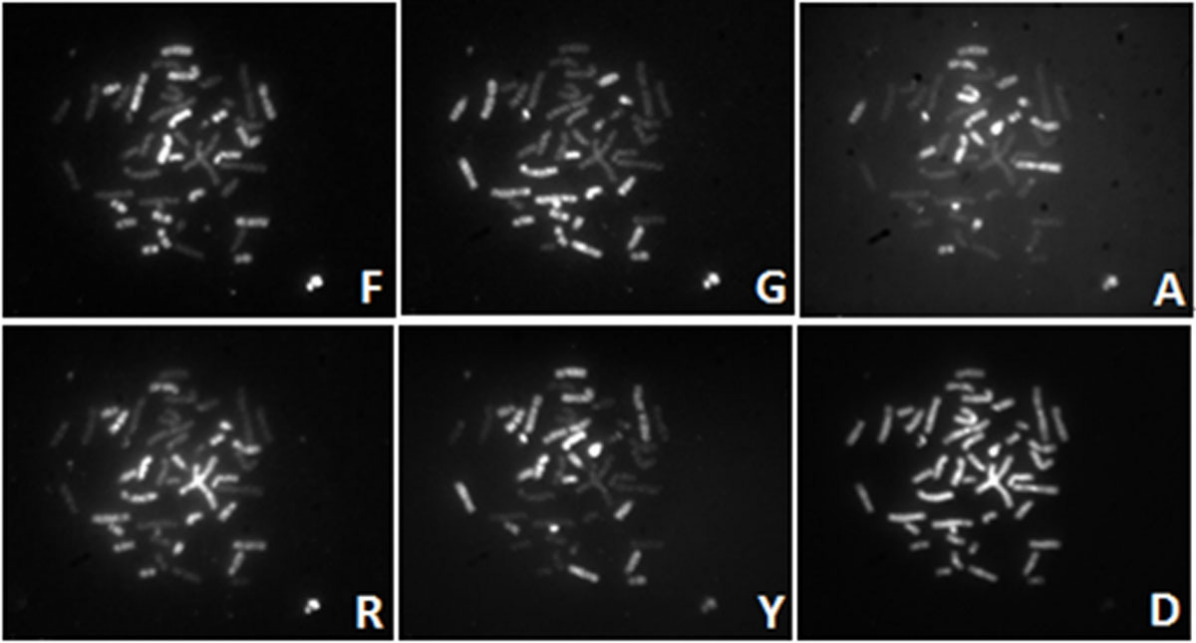
**An example of the M-FISH image set**. This figure shows the M-FISH images of a metaphase cell. There are six different channels in one set of M-FISH images. The first five channels come from different colours of florescence probing. Only some parts of the chromosomes are visible in each of these images. The last one is the DAPI channel, where all chromosomes can be observed in the cell.

Chromosomal abnormalities (e.g., changes in number and translocations of structures) could all cause genetic diseases and cancers. To detect these deathful diseases, multicolour Fluorescence *In-Situ *Hybridization (M-FISH) technique use different colours to paint human chromosomes. Therefore, this technique can be employed to analyze these abnormalities simultaneously [1,2]. This cytogenetic approach uses *N *fluorochromes to label a metaphase cell; there are *2^N^-1 *different combinations that can differentiate different types of chromosomes. It is obviously that 5 different fluorochromes are enough to differentiate 24 types of different human chromosomes. Therefore, the S Gold (F), S Green (G), S Aqua (A), Red (R) and S Red (Y) are used to paint the chromosomes. The painted chromosomes are illuminated by specific wavelength light. The fluorochromes on the chromosomes emit florescent light with distinct wavelength which can be detected by the microscopy. To acquire images of different fluorescence colours, 5 different emission filters were employed to avoid the disturbance of the other fluorescence colours and keep the valid emission light. Figure 1 illustrates M-FISH image set which is collected by microscopy with CCD camera. In addition, the last image in Figure 1 is the DAPI channel which shows the whole chromosomes in a cell. For each fluorescence channel, one image is generated and the chromosomes are detected by the pixels with high intensity. Ideally, a chromosome can be dyed with at least two fluorochromes, for example, S Green (G) and DAPI. Hence, the chromosome should be visible only in G and DAPI channels, but sometimes it might be observed in other channels because of spectral mixing, inhomogeneous background [3]. Therefore, it is extremely challenging to identify the chromosomes accurately based on M-FISH image set in practice.

For detecting the chromosomal abnormalities associated with genetic diseases or cancers by M-FISH technique, it is important to improve the accuracy of the classification of the chromosomes. Before classification, some preprocessing methods [3-7] are necessary to increase the accuracy by reducing the noise of the original images. In classification, there are two major types of classifiers: the pixel by pixel classifier [8-10] and the region-based classifier [6,7]. For the classification, we have proposed Bayesian classifier [11] and sparse representation based classification (SRC)[12]. For the segmentation purpose, we have developed Adaptive Fuzzy C-Means (AFCM) segmentation method [6]. To bring the imaging technique into clinical use, further effort is needed to improve the classification accuracy.

Sparse representation methods including compressive sensing have been widely studied recently in applied mathematics and signal/image processing for their advantages in processing high dimensional data [13,14]. There are many algorithms ( e.g., greedy algorithms (Matching Pursuit (MP [15]), OMP [16] and Homotopy [17]) to solve the sparse models. Recently Multiple Measurement Vectors (MMV) based models have also been proposed to recover a set of vectors that share a common support. Such models can find wide applications in many research fields (e.g., multiple signal classification(MUSIC)[18], blind multiband signal reconstruction[19] and compressive diffuse optical tomography[20]), where MMV problem is commonly applied. Motivated by these efforts on the MMV problem, we proposed a novel sparse representation model by incorporating the structural information into the classification of M-FISH image set, which was reported in our preliminary study [21]. This improved model considers the correlations of neighbouring pixels, which often share the same features and belong to the same class. By utilizing multiple information both from the neighbourhood of a pixel as well as from different spectral channels, the classification results of the proposed sparse model are better than that of sparse model we used before [12].

The paper is organized as follows. First, we introduce the SRC model without structural information and then propose an improved sparse model as well as the corresponding algorithm (i.e., SOMP) for estimating the solution. Next, we apply the improved model to M-FISH classification and compare it with a conventional sparse model which was employed in our previous model [12]. Finally, the paper is concluded with a short summary and discussion of the proposed model.

## Methods

The SRC model has been successfully used in many fields (e.g., hyperspectral imaging classification [22] and M-FISH chromosome classification [12]). Before introducing the improved sparse model, we first review the sparse model and show how to apply it on M-FISH image data analysis. Then, we present the improved sparse model with the structural information for M-FISH chromosome classification by utilizing correlated information of the neighbouring pixels within a region. Finally, we describe the numerical algorithm, SOMP, for solving this improved model.

### SRC algorithm for M-FISH data

**Figure 2:**
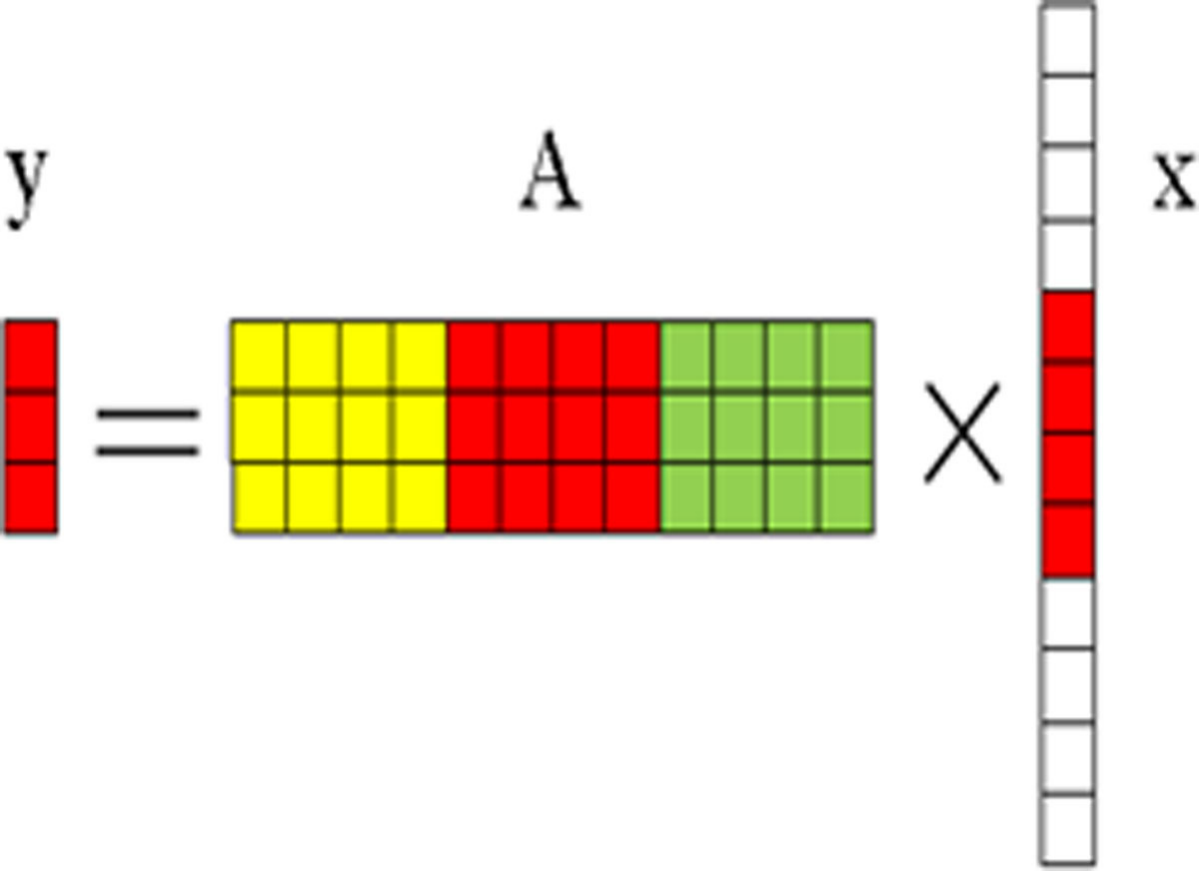
**The schematic diagram of a sparse model used for classification**. This figure shows a sparse model. A represents the feature matrix, and y is a vector to be classified. In matrix A, three different colours represent three different classes. × is a sparse vector, and has many zero entries (white colour) but a few non-zero entries (red colour) corresponding to a particular class to which y belongs.

A general type of sparse model is shown in Eq. (1), where ***y ***is a vector with different observations; ***A ***is a matrix consisting of features from different classes; and ***x ***is a vector of coefficients corresponding to the observation vector ***y***. If the observations ***y ***belongs to a particular class, the corresponding coefficients in ***x ***will have a few non-zero entries concentrated around a particular region, whereas the rest will be zeros; i.e., the vector ***x ***is a sparse vector with many zero entries. Figure 2 shows the schematic diagram of the sparse model. In Figure 2, matrix ***A ***consists of features from three different classes which are represented by different colours: yellow, red and green respectively. ***x ***is a sparse vector with non-zero entries in red region and zero entries in white regions. Given an observation vector ***y***, the sparse vector ***x ***can be solved with the optimization model shown in Eq. (2). Assuming we have *m *(i.e., m = 24 in our case) classes and each pixel corresponds to a *n *(n = 5) dimensional feature vector $a_{j,}j=1,2,...,N_{i}$, we can have a feature matrix ***A ***represented by $A=\left[A_{1},...,A_{i},...,A_{m}\right]$, where each sub-matrix is $A_{i}$ (i.e., $A_{i}=\left[a_{1},a_{2},...,a_{N_{i}}\right]$), and $A_{i}\in R^{n\times N_{i}}\;\left(N_{i}>n\right)$. Here $N_{i}$ is the number of training pixels from the *i*-th class. In matrix ***A***, the number of pixels is $N=\sum_{i=1}^{m}N_{i}$. Based on the sparse model in Eq. (1), a testing sample *y *can be approximated by a sparse solution $\hat{x}$ with non-zero coefficients corresponding to a particular class using Eq. (2).

(1) y=Ax

(2) $$\hat{x}=\underset{x}{\text{arg}\text{ min}}{∥Ax-y∥}_{2}\;\text{subject}\;\text{to}\;{∥x∥}_{p}\leq K_{0}$$

where $y\in R^{n}$ is the test pixels to be classified; ${∥x∥}_{p}$, $p\in \left[0,1\right]$ is the L-***p ***norm of ***x ***and is usually used to shrink the solution $\hat{x}\in R^{N}$ to have small percentage of nonzero coefficients, which results in the sparse of the solution; by specifying the values of K_0_,we can obtain the solution with different sparse levels. For the sake of simplicity, we take the case of p=0, and ${∥x∥}_{0}$ is the corresponding L_0 _norm of ***x***, which means the number of the non-zero coefficients in ***x***.

After estimating the solution of Esq. (1)-(2), we will classify a test sample ***y ***as follows:

(3) $$Class\left(y\right)=\text{arg }\underset{}{\text{min}}{∥y-A_{i}{\hat{x}}_{i}∥}_{2},i=1,2,...,m$$

where *m *represents the number of different classes; and ${\hat{x}}_{i}$ is the sparse solution corresponding to class *i *. The class that ***y ***belongs to is determined by assigning it to the one that the distance between the ***y ***and estimated solution $A_{i}{\hat{x}}_{i}$ is minimum.

### Improved sparse model with structural information for M-FISH data analysis

**Figure 3:**
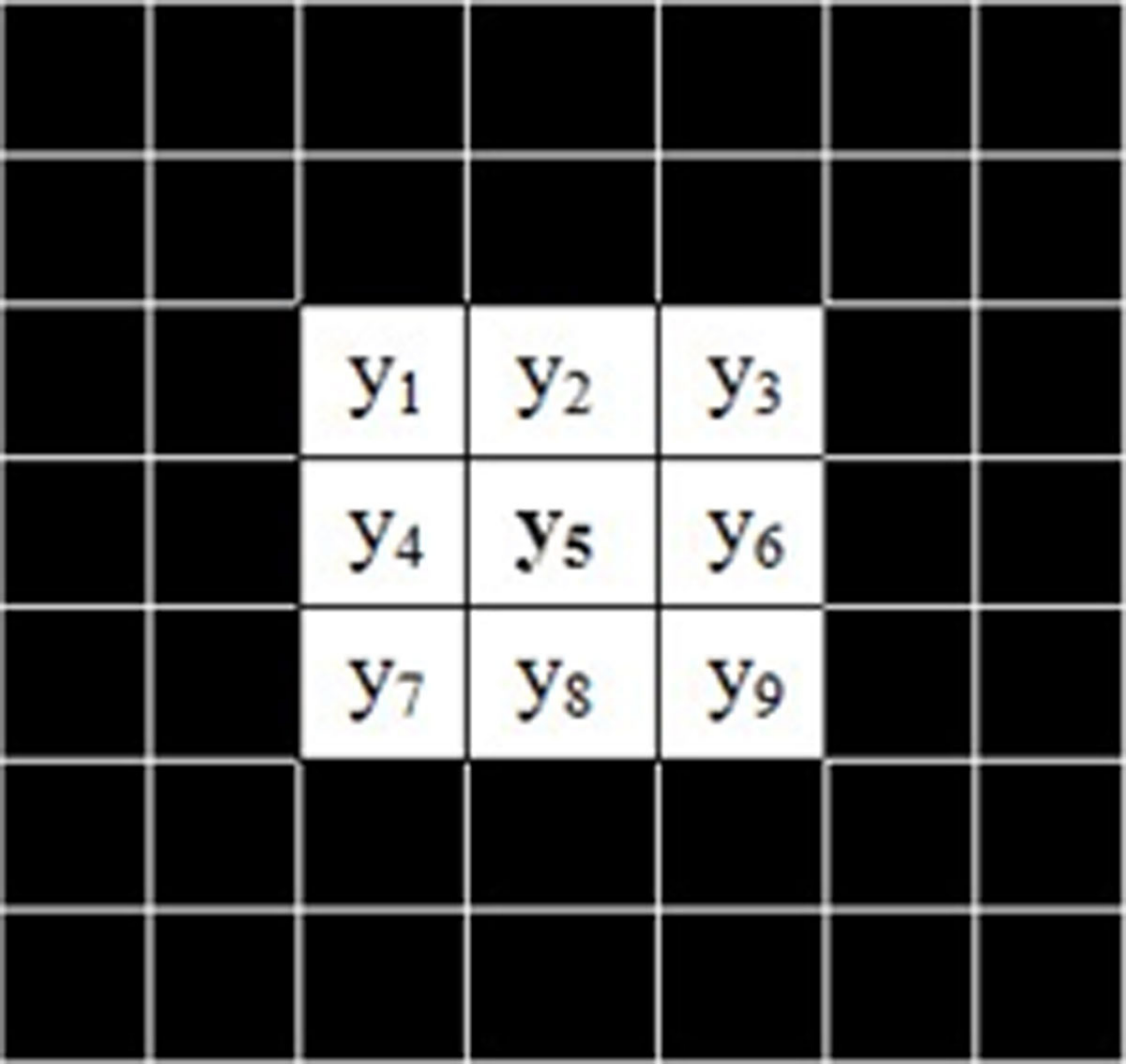
**The schematic diagram of neighbouring region**. Each square represents a pixel in an M-FISH image, corresponding to five channel spectral information. The y_5 _is a central pixel surrounded by its eight neighbouring pixels y_1_,y_2_,y_3_,y_4_,y_6_,y_7_,y_8 _and y_9_. The size of the neighbouring pixels can vary.

In the Eq. (1), ***y ***is a feature vector consisting of 5- channel spectral information at only one pixel. However, in practice a pixel usually shares the same feature with its neighbouring pixels, which is the case with M-FISH image set. The neighbouring pixels with similar intensity values are the nearest neighbourhood of ***y_5 _***which is a central pixel, as illustrated in Figure 3.

**Figure 4:**
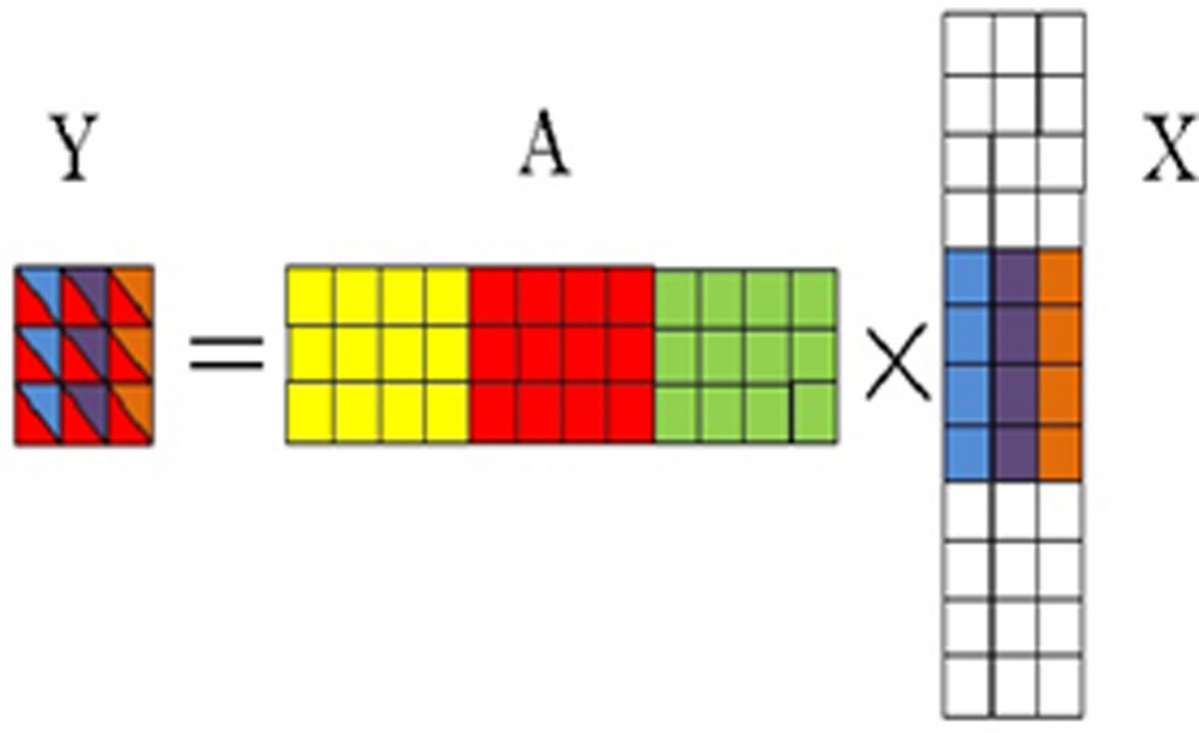
**The schematic diagram of the improved sparse model**. In matrix A, columns are features from different classes. Different colours represent different classes. Instead of using the vector x, a row-wise sparse matrix × is used, where only a few rows of × are non-zeros (the colour regions of matrix X). Each column of matrix Y is a linear combination of matrix A and the corresponding column in matrix X.

The classification accuracy of a pixel by pixel classifier and its robustness to noise can be improved by considering structural information of the pixel within a neighbour region. Therefore, we exploit a new sparse model with structural information by utilizing the information of neighbouring pixels simultaneously instead of a single pixel as shown in Eq. (4):

(4) $$\begin{array}{c} y_{1}=Ax_{1}=a_{1}x_{1,1}+a_{2}x_{1,2}+...+a_{N}x_{1,N}\\ \vdots \;\;\;\;\vdots \;\;\;\;\vdots \;\;\;\;\vdots \\ y_{9}=Ax_{9}=a_{1}x_{9,1}+a_{2}x_{9,2}+...+a_{N}x_{9,N}\\ \Rightarrow \left[y_{1}...y_{5}...y_{9}\right]=A\left[x_{1}...x_{5}...x_{9}\right]\Rightarrow Y=AX \end{array}$$

where ***y_1_***,...,***y_9_***are the test samples within a neighbourhood that form the matrix ***Y ***and ***y_5_*** is the central pixel. ***x_1_***,...,***x_9_***are the vectors of corresponding weights. Eq. (4) shows that ***y_1_***,...,***y_9 _***share the same features in matrix ***A ***but different weights. Figure 4 shows the schematic diagram of the improved sparse model with structural information based on the Eq. (4).

Since matrix ***X ***is a row-wise sparse matrix, as shown in Figure 4, the improved model is an extension of our previous sparse model (1) by considering multiple pixels simultaneously. With this improved sparse model, we propose to use the following optimization for the solution:

(5) $$\hat{X}=\text{arg}\underset{X\in R^{N\times s}}{\text{min}}{∥AX-Y∥}_{F}\;\;subject\;to\;{∥X∥}_{0,q}\leq K_{0}$$

where $Y=\left[y_{1},...y_{j},...y_{s}\right]$ is a test matrix instead of the vector in SRC model[12]. The text matrix contains *s *test pixels within a neighbouring region. Assuming that there is spatially correlated among the *s *pixels. The row-sparse solution $\hat{X}=\left[x_{1},...x_{j},...x_{s}\right]$ corresponds to the input matrix ***Y***. The entries in ${\left\{x_{j}\right\}}_{j=1,...,s}$ share the same non-zero supports. They are obtained by Eq. (5) with the following regularization term:

(6) $$l_{0,q}:\;{∥X∥}_{0,q}=\underset{i}{\sum }I\left({∥x^{i}∥}_{q}^{}>0\right)$$

where ${∥X∥}_{0,q}$ indicates the number of non-zero rows of ***X***, and $x^{i}$ indicates the *i*-th row of ***X***. $I\left({∥x^{i}∥}_{q}^{}>0\right)$ is an indicator function that has the value 1 if ${∥x^{i}∥}_{q}^{}>0$ and 0 otherwise. In this work, we set q=2. The solution vectors ${\left\{x_{j}\right\}}_{j=1,...,s}$ have the row-wise sparsity (*i.e*., the non-zero entries in the same row), which indicates the high correlation of the neighbouring pixels.

The rule of the decision used in the Eq.(3) and in the improved model is similar. After we get $\hat{X}$, we will employ Eq. (7) to determine to which class the test samples surrounding a central vector ***y_c_***belongs to,

(7) $$Class\left(y_{c}\right)=\text{arg }\text{min}{∥Y-A_{i}{\hat{X}}_{i}∥}_{F},i=1,2,...,m$$

where ***y_c _***is the central pixel of a neighbourhood and ${∥Y-A_{i}{\hat{X}}_{i}∥}_{F}$ is the residual between an input matrix ***Y ***consisting of neighbouring pixels around ***y_c_***and the product of the solution ${\hat{X}}_{i}$ and the corresponding sub-matrix ***A_i_***. The minimum value of the residual determines the class which the central pixel belongs to.

### Algorithms for the solution of the improved sparse model

There have been many approximate algorithms for solving the optimization problems (i.e., Eq.(2) and (5)). When p equals 0 [15,16], e.g., L_0 _norm, the greedy algorithms (*e.g., MP, OMP*) will be employed to solve the problem of Eq. (2). In [23], simultaneous OMP (SOMP) algorithm for Eq. (5) was employed instead of *OMP *algorithm for solve Eq. (2) and the detail of SOMP is described in Table 1. At each iteration, the algorithm will pick up one column $a_{k}$ from the training matrix ***A***based on the criterion that the maximum q-norm value of the projection on the current residual matrix could be obtained only by selecting the column $a_{k}$. Once the column is selected, it will be included for re-estimating the signal ${\hat{X}}_{i}$ and thus the new reduced residual. The algorithm will continue until the solution reaches the pre-specified sparsity level.

**Table 1:** Simultaneous Orthogonal Matching Pursuit (SOMP) algorithm

*Algorithm 1: SOMP*
**(1): Input**: training sample matrix A, testing sample matrix Y
**(2): Output**: Row-wise sparse solution $\hat{X}$
**(3): Initialization**: residual $R_{0}=Y$, ${\hat{X}}_{0}=0$, non-zero rows Ω=∅, *i *= 0
**(4): While **stopping criterion false **do**
1). Find a new atom from matrix ***A ***to best approximate the current residual based on *q*-norm: $w=\text{arg }\underset{k\in \text{Ω}}{\text{max}}{∥a_{k}^{T}R_{i-1}∥}_{q}$
2). Update the non-zero row support $\text{Ω}=\text{Ω}\cup \left\{w\right\}$.
3). Update the signal estimation ${\hat{X}}_{i}={\left(A_{\text{Ω}}^{T}A_{\text{Ω}}\right)}^{+}A_{\text{Ω}}^{T}Y$, where $A_{\text{Ω}}$ denotes the sub-matrix of A consisting of the atoms from matrix ***A***, and the residual: $R_{i}=Y-A_{\text{Ω}}{\hat{X}}_{i}$.
4)*.i = i + 1*.
**(5): End while**
**(6): Return: **$\hat{X}={\hat{X}}_{i}$

## Results and analysis

### M-FISH database

**Figure 5:**
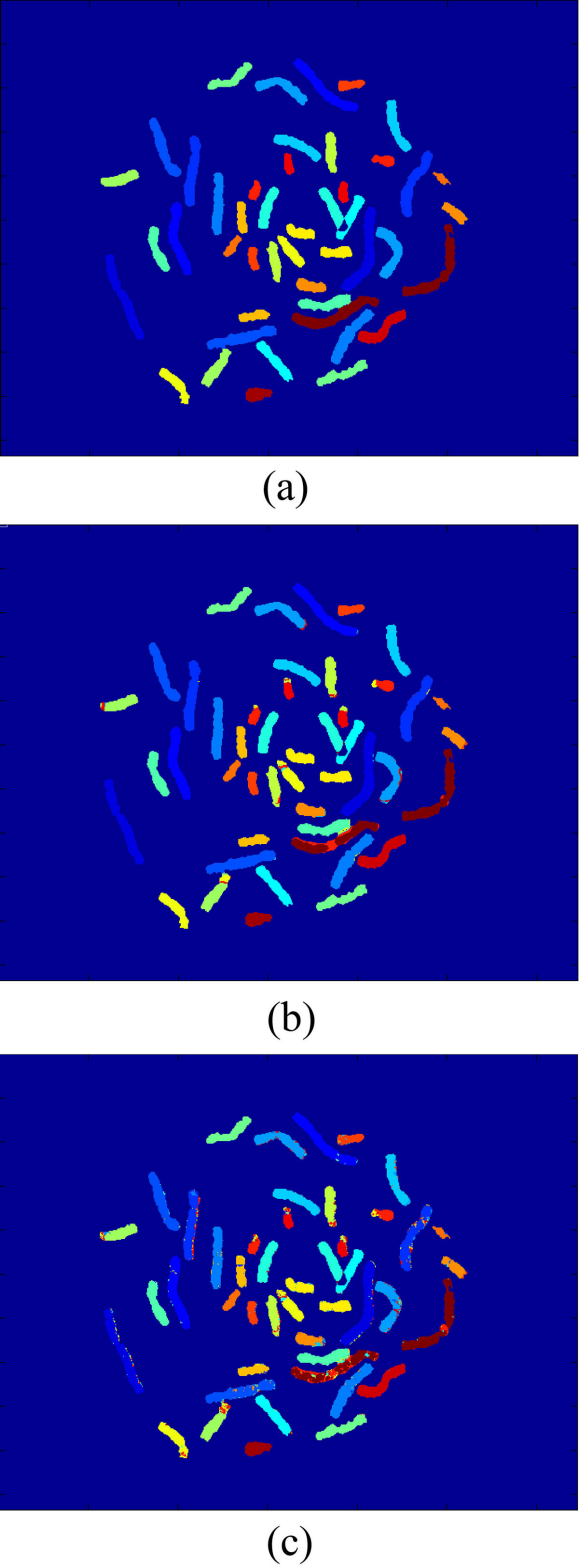
**The classification results of using two different models**. The first figure (a) is the ground truth used to verify the classification results. The second one (b) is the classification result using the improved sparse model with structural information. The third one (c) is the classification result using our previous model [12]. The classification results (i.e., types of chromosomes) are visualized in the form of pseudo colours.

We have collaborated with Advanced Digital Imaging Research (ADIR; League City, Texas, USA) to establish the M-FISH image database, which is a valuable source for chromosome imaging studies [18]. The database is publicly available from [24]. A set of images from five different fluorescence channels and a DAPI channel were acquired by microscopy and an example is shown in Figure 1. In addition, to evaluate the classification accuracy, an experienced cytogeneticist provided a ground truth image which is shown in Figure 5(a) in the form of pseudo colours, where different colours indicate different types of chromosomes. There are totally 24 different classes including male and female chromosomes. In the ground truth images, the background pixels were labelled with 0. The pixels in the region of overlap were labelled with 255. Others were labelled by numbers from 1 to 24 which was used to discriminate different types of chromosomes. The ground truth will be employed to verify the accuracy of classification algorithms for M-FISH image set.

### Segmentation of chromosome regions

**Figure 6:**
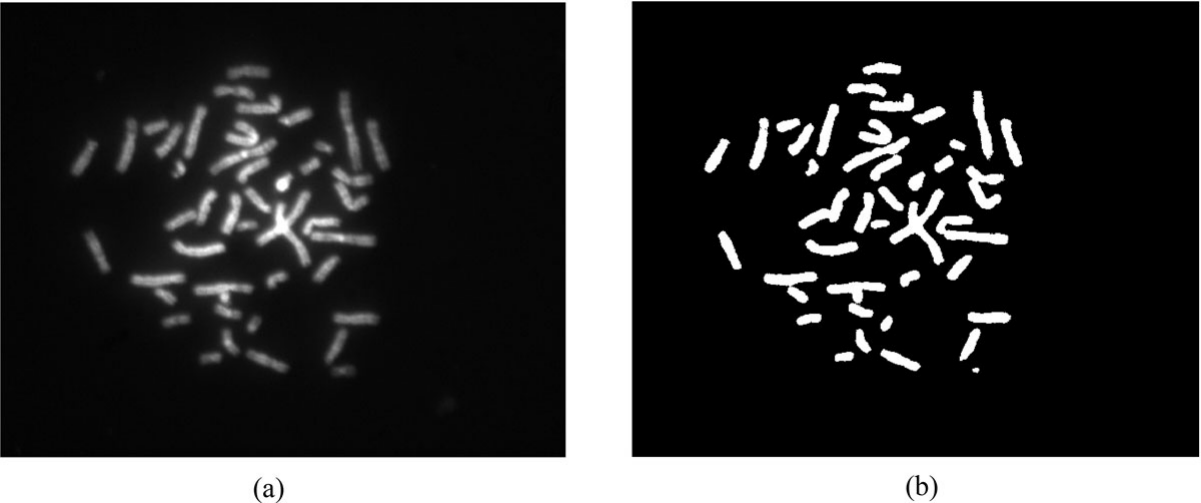
**An example of a DAPI channel and the segmented mask**. Figure (a) is the DAPI channel of an M-FISH image set. Figure (b) is the result of the segmentation by AFCM method. This segmentation result was used as the mask for other five channels and the chromosome classification is only carried out in this mask region.

In M-FISH images, background usually contains most pixels, but the chromosomal regions are of most interests. Therefore, to separate the chromosomal region from the background and improve the efficiency of the classification, a mask was generated by the DAPI channel which can show all chromosomes in a cell. The AFCM method we proposed in [5] was employed for this purpose. This mask was then applied on the other five channels, so that the chromosome regions could be extracted based on the mask while the pixels out of the mask were removed. In Figure 6, an image of a DAPI channel is demonstrated as well as how the mask is generated by the segmentation.

### M-FISH training and testing data

The improved sparse model with structural information was applied on the classification of M-FISH image data. 20 cells (i.e., 10 male, 10 female) were chosen from our database [24]. The features of different types of chromosome were constructed by randomly sampling pixels from M-FISH images to form the training matrix ***A***, which satisfy the sparsity concentration index (SCI) proposed by[25]. SCI is used to measure the sparsity concentration of the feature vectors. Matrix ***A ***is an *n×N *matrix, in which *n *represents the spectral dimension of pixels and *N *represents the number of training features. In the case of M-FISH image data, *n *equals 5. After completing the matrix training, the rest of the pixels were taken as testing data to validate our proposed classification method.

### The analysis of the classification results with different models

Both the sparse model incorporating the structural information and our previously used sparse model [12] were tested and compared on our M-FISH data set. Figure 5(b) and 5(c) show the classification results of two different models on the same cell, with and without the use of structural information respectively. It can be seen that there are more isolated spots in the chromosomal regions of Figure 5 (c) than those of Figure 5 (b). These isolated spots are mostly misclassifications, which can be effectively corrected by using the improved sparse model with structural information. The ratio of correct classification (RCC) as follow:

(8) $$RCC=\frac{\text{the ratio of the number of}\;\text{correctly classified pixels}}{\text{the number of all}\;\text{pixels in a chromosomal region}}$$

**Figure 7:**
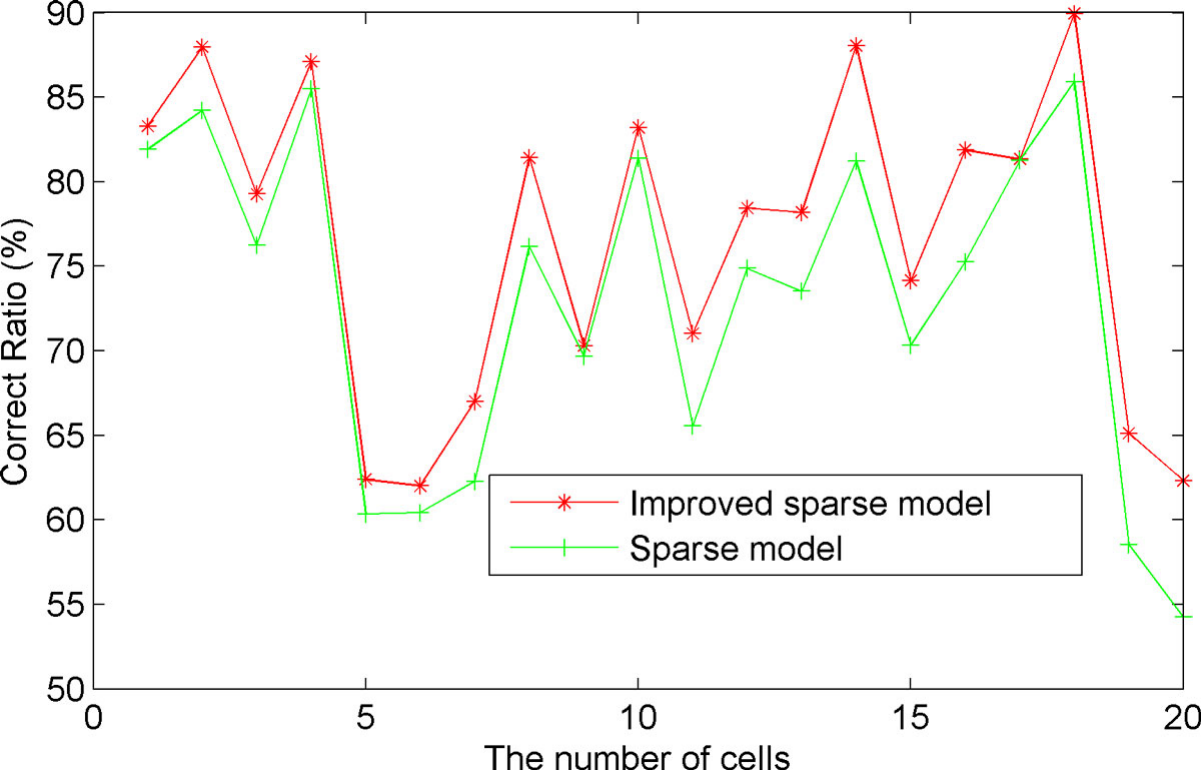
**The RCC of two models**. The red line is the RCC of using the new sparse model incorporating the structural information for 20 different cells. The green line is the RCC of using our previous model (i.e., general sparse model) for the same 20 cells.

Table 2 shows RCC of different types of chromosomes for one M-FISH image set. The RCC of the improved sparse model with structural information is generally greater than that of our previously used sparse model. Figure 7 compares the classification results of both models on each cell in terms of RCC. It can be seen that the accuracy of the classification of the improved sparse model with structural information (in red) is greater than that of the previously used sparse model [12] (in blue). Therefore, with the structural information of neighbouring region, the improved sparse model can increase the accuracy of the classification for the M-FISH image set.

**Table 2:** The correct classification ratio of each class in an M-FISH image

Class number	New sparse model	General sparse model	Class number	New sparse model	General sparse model
1	0.951282	0.894359	13	0.903226	0.895439
2	0.988194	0.961629	14	0.832192	0.785388
3	0.930451	0.929825	15	0.969388	0.926304
4	0.972441	0.919948	16	1	0.993921
5	0.983595	0.905136	17	1	0.998273
6	0.975627	0.965181	18	0.929553	0.917526
7	0.967769	0.953719	19	1	0.977175
8	0.959322	0.881356	20	0.930556	0.882937
9	0.997059	0.978431	21	0.832817	0.758514
10	1	0.991313	22	0.997361	0.960422
11	0.958773	0.967402	23	0.981279	0.976599
12	0.997038	0.99309	24	1	0.990506

### Significance analysis of the new sparse model with structural information

**Figure 8:**
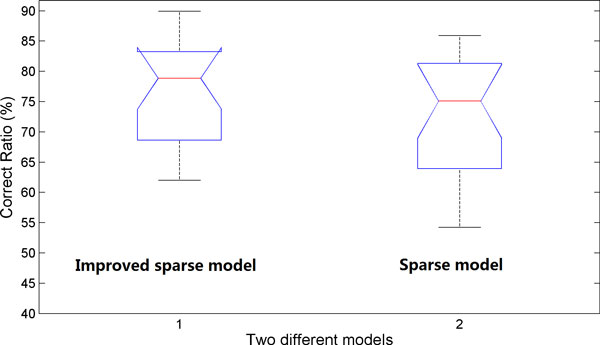
**Statistical analysis of two different sparse models based classification methods**. The first box plot shows the quartiles and ranges of the classification results of the new sparse model incorporating the structural information, while the second one shows those using our previous model.

Statistical analysis by using a paired-sample t-test was performed to demonstrate the significant level between the two different models. The null hypothesis is that there are no differences between both models. Figure 8 shows the results of the statistical analysis based on the results in Figure 7. The improved sparse model with structural information has the greater mean value while less standard deviation, 76.72 ± 9.3 (i.e., the left box plot in Figure 8), than those of the previous sparse model, 72.94 ± 9.82 (i.e., the right box plot in Figure 8). The significant level (i.e. p-value) of this statistical analysis is less than 1e-6. Therefore, the improved sparse model with the structural information significantly outperforms our previous sparse model, by incorporating the structural information available in the neighbour of each pixel.

### Effects of parameters used

**Figure 9:**
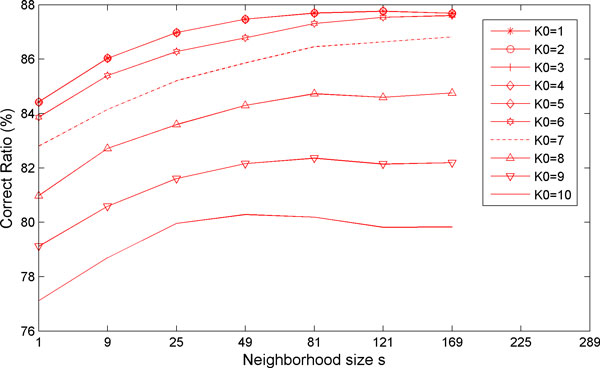
**The effect of the *K_0 _*and *s *on the RCC**. Different lines represent the accuracy of classifying an M-FISH image for different sparsity level (K_0_) and different neighbourhood size s. K_0 _is changed from 1 to 10 and s is changed from 1 to 169. When K_0 _equals 1 to 5, there will be almost no difference among different neighbourhood size s. However, when K_0 _is greater than 5, the RCC will change dramatically with different sparsity level.

There are three important parameters, neighbour size (*s*), sparsity level (*K_0_*), and training sample size (*N_i_*), which are involved in the improved sparse model. The accuracy of the classification results can be affected by these three parameters and hence it is worthwhile to study their effects. Figure 9 shows how the RCC is affected by different values of *K_0 _*and *s*. When *K_0 _*is fixed, the RCC will raise with the increase of the neighbourhood size *s *until a certain threshold (e.g., *s *= 121). This indicates that the use of correlated information within a window can generally increase the classification accuracy, however, if the window size is too large, there is high probability that more irrelevant or other chromosomal pixels will be included, which tends to increase the classification error. An appropriate window size is therefore needed. A neighbourhood size (*s *= 9) is recommended based on our experiments. When the neighbourhood size *s *is fixed, from Figure 9, the smaller value of the sparsity level *K_0 _*will give the greater accuracy of the classification.

**Figure 10:**
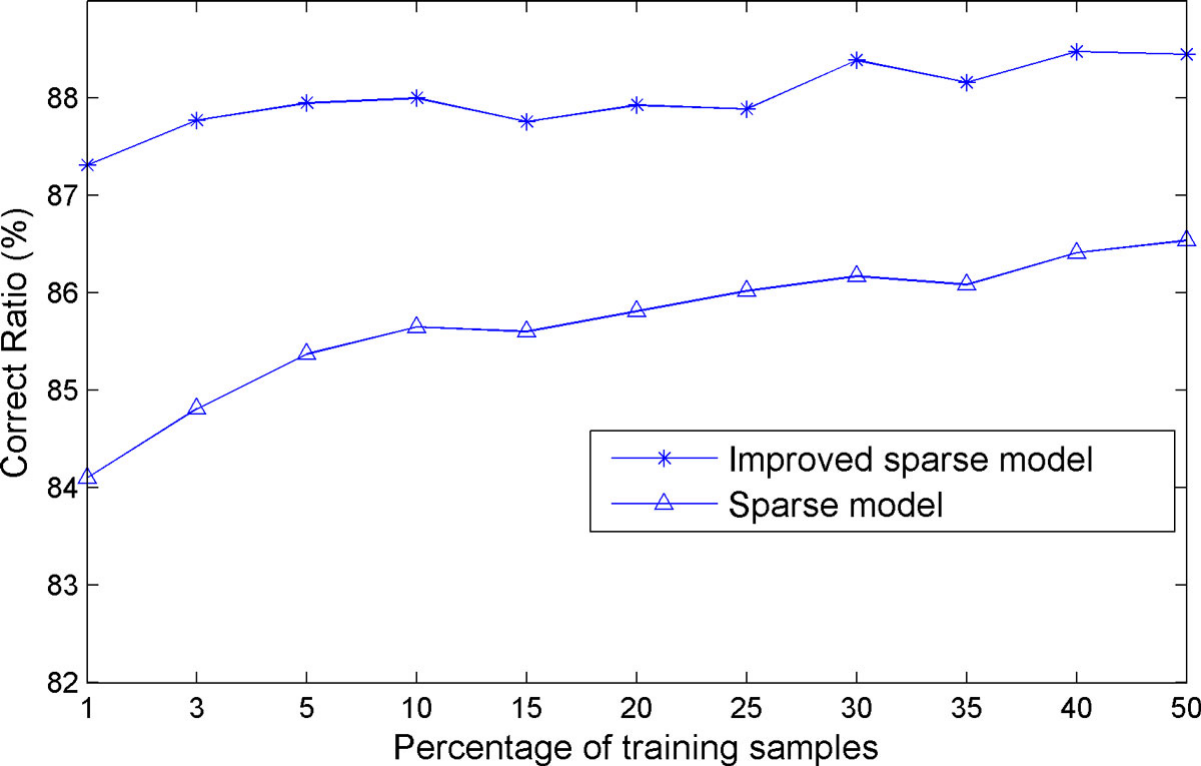
**The effect of the *N_i _*on the RCC**. The star line represents the RCC of the new sparse model incorporating the structural information with respect to different percentage of training samples, whereas the triangle line represents the RCC of our previous model [12]. The results of the improved sparse model are much better than those of our previous model. In addition, with the increase of the training samples, the classification ratio increases.

In addition, the correct ratio of classifying the M-FISH image is affected by the training sample size *N_i_* for both models as shown in Figure 10. A number of different percentages of training samples were selected: 1%, 3%, 5%, 10%, 15%, 20%, 25%, 30%, 35%, 40%, and 50%. In Figure 10, the correct classification ratios of the two models are represented by stars and triangles respectively. The analysis results show that the correct classification ratios increase with the increasing size of the training samples for both models, which is reasonable.

## Conclusions and discussion

A sparse model based classifier that we proposed before [12] used the pixel by pixel classification, overlooking structural information so that there are much more isolated spots in the results leading to the low accuracy of the classification. In this paper we proposed an improved sparse model, in which the information of a central pixel as well as its neighbouring pixels is used simultaneously for improved classification. This is validated by the comparison of chromosomal classification accuracy between the two models on a real M-FISH database [24]. The comparison (as illustrated by Figure 5) shows that there are more isolated spots (i.e., misclassifications) in the classification results of our previously model [12] than those of using new sparse model incorporating the structural information. The correct classification ratio in Table 2 also shows the improved accuracy of using the improved sparse model. The statistical comparison between the two models indicates that the new sparse model with structural information is superior to the previously used sparse model, with the significant level less than 1e-6,. The effects of parameters used in the model on the accuracy of classification were also investigated. We have shown how the sparsity level (*K_0_*) and the neighbourhood size (*s*) and the training sample size *(N_i_) *affected the RCC of our improved sparse model incorporating structural information and how the training sample size *(N_i_) *affected the RCC of our previously used model as well as improved model. A proper choice of sparsity level (*K_0_*< = 5) and neighbourhood size (*s *= 9) is recommended based on our experiments.

In summary, all the result shows that our proposed improved sparse model incorporating structural information can significantly improve the accuracy of the classification compared with a general sparse model that we proposed before [12]. This will in turn improve the M-FISH imaging technique for detecting chromosome abnormalities to better diagnose genetic diseases and cancers.

## Competing interests

The authors declare that they have no competing interests.

## Authors' contributions

JL, DL and YPW designed research. JL designed the algorithm. HC performed segmentation algorithm. All authors read and approved the final manuscript.

## Declarations

The publication costs for this article were funded by the corresponding author.

This article has been published as part of *BMC Systems Biology *Volume 7 Supplement 4, 2013: Selected articles from the IEEE International Conference on Bioinformatics and Biomedicine 2012: Systems Biology. The full contents of the supplement are available online at http://www.biomedcentral.com/bmcsystbiol/supplements/7/S4.

## References

[B1] Schrock E duManoir S Veldman T Schoell B Wienberg J FergusonSmith MA Ning Y Ledbetter DH BarAm I Soenksen D Multicolor spectral karyotyping of human chromosomes Science 1996 7 5274 494 497 10.1126/science.273.5274.494 8662537

[B2] Speicher MR Ballard SG Ward DC Karyotyping human chromosomes by combinatorial multi-fluor FISH Nat Genet 1996 7 4 368 375 10.1038/ng0496-368 8630489

[B3] Choi H Bovik AC Castleman KR Feature normalization via expectation maximization and unsupervised nonparametric classification for M-FISH chromosome images IEEE Trans Med Imaging 2008 7 8 1107 1119 18672428 10.1109/TMI.2008.918320

[B4] Choi H Castleman K Bovik A Joint segmentation and classification of M-FISH chromosome images Conf Proc IEEE Eng Med Biol Soc 2004 7 1636 1639 17272015 10.1109/IEMBS.2004.1403495

[B5] Cao HB Deng HW Wang YP Segmentation of M-FISH Images for Improved Classification of Chromosomes With an Adaptive Fuzzy C-means Clustering Algorithm Ieee T Fuzzy Syst 2012 7 1 1 8

[B6] Karvelis PS Fotiadis DI Tsalikakis DG Georgiou IA Enhancement of multichannel chromosome classification using a region-based classifier and vector median filtering IEEE Trans Inf Technol Biomed 2009 7 4 561 570 19171531 10.1109/TITB.2008.2008716

[B7] Karvelis PS Tzallas AT Fotiadis DI Georgiou I A multichannel watershed-based segmentation method for multispectral chromosome classification IEEE Trans Med Imaging 2008 7 5 697 708 18450542 10.1109/TMI.2008.916962

[B8] Sampat ACB MP Aggarwal JK Castleman KR Pixel-by-pixel classification of MFISH images24th IEEE Ann Intern Conf (EMBS) 2002 7 Houston, TX 999 1000

[B9] Schwartzkopf WC Bovik AC Evans BL Maximum-likelihood techniques for joint segmentation-classification of multispectral chromosome images Ieee T Med Imaging 2005 7 12 1593 1610 16350919 10.1109/TMI.2005.859207

[B10] Sampat MP Bovik AC Aggarwal JK Castleman KR Supervised parametric and non-parametric classification of chromosome images Pattern Recogn 2005 7 8 1209 1223 10.1016/j.patcog.2004.09.010

[B11] Wang YP Castleman KR Normalization of multicolor fluorescence in situ hybridization (M-FISH) images for improving color karyotyping Cytom Part A 2005 7 2 101 109 10.1002/cyto.a.20116 15729736

[B12] Cao HB Deng HW Li M Wang YP Classification of Multicolor Fluorescence In Situ Hybridization (M-FISH) Images With Sparse Representation Ieee T Nanobiosci 2012 7 2 111 118 22665392 10.1109/TNB.2012.2189414 PMC4165853

[B13] Simoncelli EP Olshausen BA Natural image statistics and neural representation Annu Rev Neurosci 2001 7 1193 1216 10.1146/annurev.neuro.24.1.1193 11520932

[B14] Li Y Cichocki A Amari S Analysis of sparse representation and blind source separation Neural Comput 2004 7 6 1193 1234 10.1162/089976604773717586 15130247

[B15] Mallat SG Zhang ZF Matching Pursuits with Time-Frequency Dictionaries Ieee T Signal Proces 1993 7 12 3397 3415 10.1109/78.258082

[B16] Tropp JA Gilbert AC Signal recovery from random measurements via orthogonal matching pursuit Ieee T Inform Theory 2007 7 12 4655 4666

[B17] Donoho DL Tsaig Y Fast Solution of l(1)-Norm Minimization Problems When the Solution May Be Sparse Ieee T Inform Theory 2008 7 11 4789 4812

[B18] Kim JM Lee OK Ye JC Compressive MUSIC: Revisiting the Link Between Compressive Sensing and Array Signal Processing IEEE T Inform Theory 2012 7 1 278 301

[B19] Mishali M Eldar YC Blind Multiband Signal Reconstruction: Compressed Sensing for Analog Signals IEEE T Signal Proces 2009 7 3 993 1009

[B20] Lee O Kim JM Bresler Y Ye JC Compressive diffuse optical tomography: noniterative exact reconstruction using joint sparsity IEEE Trans Med Imaging 2011 7 5 1129 1142 21402507 10.1109/TMI.2011.2125983

[B21] Li J Lin D Cao H Wang Y Classification of multicolor fluorescence in-situ hybridization (M-FISH) image using structure based sparse representation model Bioinformatics and Biomedicine (BIBM), 2012 IEEE International Conference on: 4-7 October 2012 2012 1 6 10.1109/BIBM.2012.6392672

[B22] Chen Y Nasrabadi NM Tran TD Hyperspectral Image Classification Using Dictionary-Based Sparse Representation IEEE T Geosci Remote 2011 7 10 3973 3985

[B23] Tropp JA Gilbert AC Strauss MJ Algorithms for simultaneous sparse approximation. Part I: Greedy pursuit Signal Process 2006 7 3 572 588 10.1016/j.sigpro.2005.05.030

[B24] M-Fish Database website https://sites.google.com/site/xiaobaocao006/database-for-download

[B25] Wright J Yang AY Ganesh A Sastry SS Ma Y Robust Face Recognition via Sparse Representation Ieee T Pattern Anal 2009 7 2 210 227 19110489 10.1109/TPAMI.2008.79

